# Overcoming the Challenges of Phytochemicals in Triple Negative Breast Cancer Therapy: The Path Forward

**DOI:** 10.3390/plants12122350

**Published:** 2023-06-16

**Authors:** Mohammed Alaouna, Clement Penny, Rodney Hull, Thulo Molefi, Nkhensani Chauke-Malinga, Richard Khanyile, Malose Makgoka, Meshack Bida, Zodwa Dlamini

**Affiliations:** 1 SAMRC Precision Oncology Research Unit (PORU), DSI/NRF SARChI Chair in Precision Oncology and Cancer Prevention (POCP), Pan African Cancer Research Institute (PACRI), University of Pretoria, Pretoria 0001, South Africa; rodney.hull@up.ac.za (R.H.); thulo.molefi@up.ac.za (T.M.); chauke-malinga@up.ac.za (N.C.-M.); richard.khanyile@up.ac.za (R.K.); malose.makgoka@up.ac.za (M.M.); meshack.bida@nhls.ac.za (M.B.); 2 Department of Internal Medicine, School of Clinical Medicine, Faculty of Health Sciences, University of the Witwatersrand, Parktown 2193, South Africa; clement.penny@wits.ac.za; 3 Department of Medical Oncology, Steve Biko Academic Hospital and University of Pretoria, Pretoria 0001, South Africa; 4 Department of Plastic and Reconstructive Surgery, Faculty of Health Sciences, Steve Biko Academic Hospital, University of Pretoria, Pretoria 0001, South Africa; 5 Department of Surgery, Faculty of Health Sciences, Steve Biko Academic Hospital, University of Pretoria, Pretoria 0001, South Africa; 6 Department of Anatomical Pathology, National Health Laboratory Service (NHLS), University of Pretoria, Pretoria 0001, South Africa

**Keywords:** phytomedicine, tyrosine kinase, Wnt, epithelial to mesenchymal transition, angiogenesis, metastasis, apoptosis

## Abstract

Triple negative breast cancer (TNBC) is a very aggressive subtype of breast cancer that lacks estrogen, progesterone, and HER2 receptor expression. TNBC is thought to be produced by Wnt, Notch, TGF-beta, and VEGF pathway activation, which leads to cell invasion and metastasis. To address this, the use of phytochemicals as a therapeutic option for TNBC has been researched. Plants contain natural compounds known as phytochemicals. Curcumin, resveratrol, and EGCG are phytochemicals that have been found to inhibit the pathways that cause TNBC, but their limited bioavailability and lack of clinical evidence for their use as single therapies pose challenges to the use of these phytochemical therapies. More research is required to better understand the role of phytochemicals in TNBC therapy, or to advance the development of more effective delivery mechanisms for these phytochemicals to the site where they are required. This review will discuss the promise shown by phytochemicals as a treatment option for TNBC.

## 1. Introduction

Breast cancer is a common disease and is the main cause of cancer-related mortality in women worldwide. According to the American Cancer Society, 2.3 million new instances of breast cancer and 685,000 fatalities were reported in 2020 [1,2]. Breast cancer incidence varies greatly between nations, with estimates ranging from 40 to more than 80 incidences per 100,000 women [3]. The disparity is predicted to widen in the coming years, with an estimated three million more cases and one million more deaths expected to occur by 2040 [4,5].

Triple negative breast cancer (TNBC) is a subtype of breast cancer that accounts for 10–20% of all incidences [6,7]. TNBC is distinguished by the lack of expression of oestrogen, progesterone, and HER2 receptors, and is noted for its aggressiveness and poor prognosis [8,9]. TNBC is associated with genetic changes in genes such as TP53 and BRCA1/2 [10], and it is more common in certain ethnic groups, such as African American women and Ashkenazi Jewish women [11,12]. TNBC also has a poorer 5-year survival rate than other subtypes of breast cancer, according to the National Cancer Institute [13,14].

Chemotherapy is the primary treatment for TNBC, with regimens often including anthracyclines and taxanes [15,16,17]. TNBC treatment options are restricted, since standard hormone therapy and targeted medicines have been unsuccessful [18,19]. Despite recent advances in immunotherapy and customized treatment strategies for TNBC, the illness remains difficult to treat and additional effective treatments are required.

## 2. Risk Factors for Triple Negative Breast Cancer (TNBC)

TNBC is a subtype of breast cancer that has a more aggressive course and a worse prognosis than other subtypes. To develop suitable treatment choices and enhance patient outcomes, it is vital to understand the multiple hereditary and environmental risk factors that contribute to the development of TNBC. Understanding these traits is crucial for improving the outcomes of TNBC patients.

### 2.1. Hereditary Factors

TNBC development is influenced by hereditary factors. Individuals with a BRCA1 mutation are more likely to develop TNBC [20,21,22]. Other genetic variants in genes, including TP53 and PIK3CA, have been associated with an increased risk of TNBC [23,24]. Breast cancer in the family, particularly TNBC, has been associated with a higher risk of developing the disease. Furthermore, African Americans and Ashkenazi Jewish women are more likely to develop TNBC and have a poorer prognosis than those of other ethnicities [11,25]. The increased risk for African American and Ashkenazi Jewish women to develop triple negative breast cancer (TNBC) is thought to be due to a combination of hereditary and environmental variables.

Mutations in BRCA1 are more frequent in Ashkenazi Jewish communities and studies have indicated that these mutations increase the risk of TNBC [6,26]. This is only one of many genetic variants that can contribute to the development of TNBC. Identifying mutations that predispose individuals to TNBC, and that are unique to specific ethnicities, is complicated by socioeconomic and cultural issues, such as poor access to healthcare. These factors may disproportionally affect specific communities and may contribute to greater TNBC prevalence in, for instance, African American women [11,27,28]. More research is needed to completely comprehend the contribution of genetic differences to these ethnic groups.

### 2.2. Environmental Risk Factors

Environmental factors, in addition to genetic ones, have a role in the development of TNBC. Age influences the development of TNBC: older women with TNBC have a better prognosis and longer 6-year survival than younger women, for reasons that are as yet unknown [29]. It has been proposed by some that tumors in older women develop more slowly, or that the tumor microenvironment in older women is less favorable to tumor growth and dissemination. Furthermore, older women may have a more mature immune system as well as a larger proportion of immune cells capable of identifying and fighting cancer cells, which may contribute to better outcomes and increased survival. While older women with TNBC may have a better overall prognosis, individual cases can vary substantially, and factors such as general health and comorbidities can have a major influence on the outcomes. Hormonal changes that occur with age, such as decreased oestrogen levels, may also play a role in the better outcome in older women [30].

Obesity, defined as extra weight or fat, raises the risk of TNBC [31]. Obesity increases the risk of TNBC by causing hormonal imbalances, inflammation, and insulin resistance [32,33]. These biochemical alterations have been linked to an increased risk of getting cancer, particularly TNBC. Furthermore, adipokines, which are signaling molecules generated by fat cells, may have a role in raising the risk of TNBC [34]. Hormonal treatment, which includes hormone replacement therapy and oral contraceptives, is another concern [7,35]. Exposure to radiation, such as that which a patient is exposed to during medical imaging, has also been linked to an increased incidence of TNBC [36].

## 3. Triple Negative Breast Cancer (TNBC) Signaling Pathways

The molecular mechanisms behind TNBC remain unclear despite the fact that several signaling pathways have been identified as playing a role in the development and progression of TNBC. The epidermal growth factor receptor (EGFR) pathway [37,38], the PI3K/Akt pathway [39,40,41], the RAS/RAF/MEK/ERK network [42,43], the Wnt/β-catenin pathway [44,45], and the cyclooxygenase-2 (COX-2) pathway are among the most well-studied signaling pathways associated with TNBC [46,47]. These signaling pathways implicated in cancer are known to be targeted by various phytochemicals that show evidence of targeting these molecular pathways, including curcumin, resveratrol, green tea polyphenols, sulforaphane, erucin, genistein, genipin, baicalein, quercetin, isoquercitin, vitamin E, parthenolide, dioscin, triptolide, kaempferol, pterostilbene, isoliquiritigenin, and escin [48].

### 3.1. EGFR Pathway

Tyrosine kinase receptors are growth-factor-binding cell surface receptors. When a growth factor binds to the receptor, the receptor’s kinase domain is activated, resulting in tyrosine phosphorylation and intracellular signaling [49]. The EGF receptor tyrosine kinase family is one of the best-studied receptor tyrosine kinase families (also known as the ErbB receptor family). These receptors bind to a variety of growth factors, including EGF. Many signal transduction pathways are activated by ErbB receptors, including the RAS/Erk pathway, the PI3K/AKT pathway, and the JAK/STAT system [50]. Additionally, ErbB receptors have the ability to translocate to the nucleus and initiate transcription [51].

The EGFR pathway is activated when a ligand binds to the EGFR, causing receptor dimerization and autophosphorylation. This activates downstream signaling pathways that drive cell proliferation, survival, and migration, such as the RAS/RAF/MEK/ERK and PI3K/Akt pathways [52,53,54]. TNBC cells have been found to overexpress EGF, which has been associated with a poor prognosis [37,38]. The PI3K/Akt signaling pathway is required for cell survival and growth. When this pathway is triggered, Akt is phosphorylated, activating a number of downstream targets involved in cell survival and proliferation [55,56]. Changes in the PI3K/Akt pathway have been associated with TNBC carcinogenesis and resistance to therapy [39,40,41]. The RAS/RAF/MEK/ERK signaling system is a critical signaling pathway that regulates cell growth and survival. The downstream kinases, MEK and ERK, are phosphorylated and activated when this pathway is active [57,58]. The activation of this pathway is upregulated in TNBC and is associated with a poor prognosis [52,53,54].

Molecular docking studies and simulations have been used to study the interaction between EGFR and a multitude of phytochemicals. These include ethanol-soluble phytochemicals from *Arnica montana* such as caryophyllene oxide and hydroxycadalene c [59], furanocoumarin [60] and PH-1 (4-methyl-5-oxo-tetrahydrofuran-3-yl acetate), and PH-2 (methyl 4-hydroxy-3-methoxybenzoate) from *Polygonum hydropiper* [61]. All of these compounds were found to dock with the EGFR receptor with high affinity. Cruciferous vegetables contain the phytochemical diindolylmethane (DIM). In immunofluorescence studies, treatment of cells with DIM led to a decrease in the expression of EGFR. It also blocked ligand binding to the EGF receptor and inhibited downstream Ras-mediated PI3K-Akt-mTOR signaling [62]. In addition, DIM showed activity against drug-resistant breast cancer cells that expressed mutant isoforms of EGFR [63].

**Figure 1:**
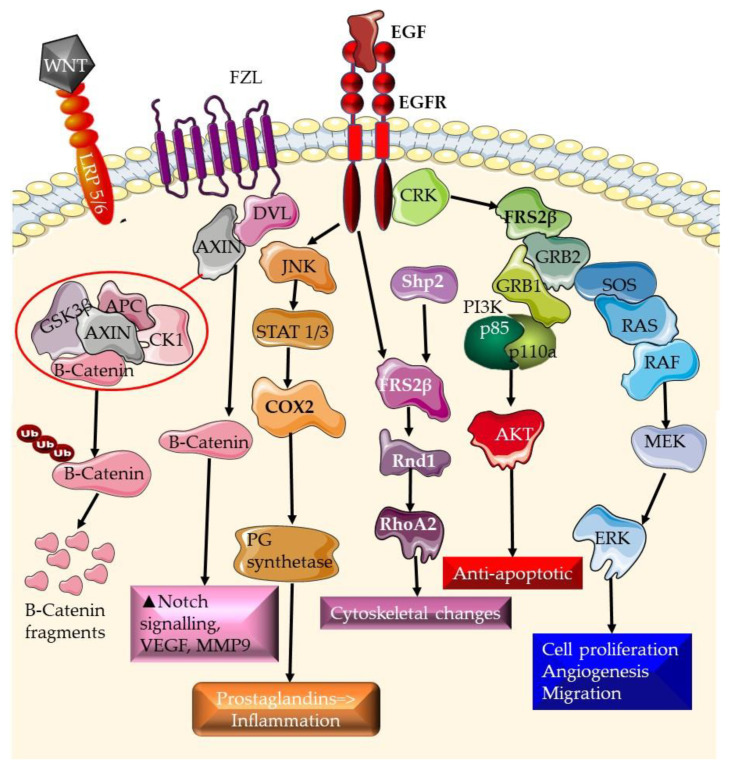
Tyrosine kinase (EGF pathway) and Wnt/β-catenin pathways. The separate pathways that control cell proliferation, cell survival, metastasis, angiogenesis, and inflammation that can be targeted by phytochemicals. The Wnt signaling pathway controls the activation of the Notch signaling pathway and controls the expression of the VEGF pro-angiogenic growth factor. The EGF and other receptor tyrosine kinase signaling activates Cox pathways, which promote inflammation through the production of prostaglandins. This results in the activation of the AKT protein, leading to pro-survival factors, inhibiting apoptosis. Finally, the ERK and MAPK pathways lead to the activation of cell migration, induction of cell proliferation, and angiogenesis.

**Figure 2:**
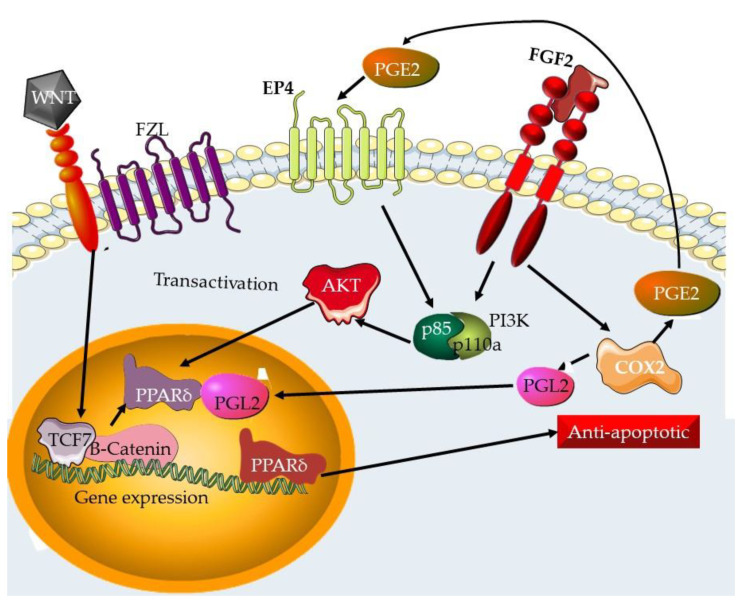
Overlap between the tyrosine kinase pathway, Wnt/β-catenin, and prostaglandin inflammation pathways. The first point of overlap is the Cox protein, which is activated through receptor tyrosine kinase signaling, then the resulting prostaglandins, which activate the EP4 transmembrane receptor, which in turn can activate the AKT pathway leading to the transactivation of the PPARδ transcription factor. The expression of PPARδ is activated by the Wnt signaling pathway. This transcription factor promotes survival by inhibiting apoptosis.

The COX-2 pathway is involved in the generation of prostaglandins and has been related to cancer development and progression [64,65]. COX-2 signaling is initiated by RTK pathways (Figure 1), where it results in the generation of prostaglandins and is responsible for inflammation responses. COX-2 overexpression has been associated with a poor prognosis in numerous malignancies, including TNBC [46,47]. COX-2 signaling is a component of the EGFR, RTK, and the Wnt signaling pathways (Figure 2). Here, COX-2 generates prostaglandins.

The phytochemical honokiol, isolated from the Magnolia species., is able to inhibit migration and the molecular mechanisms underlying cancer in breast cancer cells. Apart from inflammation, COX-2 activity is associated with migration because transfection of breast cancer cells with COX-2 siRNA results in decreased cell migration. It was proposed that honokiol inhibited the upstream promoter of COX-2, nuclear factor κB (NF-κB) [66]. Anti-inflammatory assays and computer docking models identified 77 phytochemicals that have anti-inflammatory activity with the ability to bind to COX-2. Three of these compounds, *N-*(2-hydroxycyclohexyl)-4-methylbenzenesulfonamide, Benzeneethanamine, 2-fluoro-. beta., 3, 4-trihydroxy-*N*-isopropyl, and 3,5-di-tert-butylphenol have a better binding affinity for COX-2 than the conventional anti-inflammatory drug naproxen [67]. Both ethanol and water-soluble extracts from *Parinari kerstingii* showed anti-inflammatory activity in a carrageenan-induced paw oedema rat model. This resulted in reduced swelling of the model rats’ paws. This was accompanied by a decrease in the expression of TNF-α, COX-2, and NF-κB [68]. Phenophases extracted from *Thymus longicaulis* C. Presl. Lamiaceae contain low molecular-weight phenols and flavonoids. One of the main compounds, rosmarinic acid, was shown to have anti-inflammatory, cytotoxic, and antioxidant activities against MDA-MB-231 TNBC cell lines. The activity of the extract is due to the ability of rosmarinic acid to inhibit the expression of COX-2 [69].

### 3.2. Wnt/β-Catenin Pathway

The Wnt/β-catenin signaling pathway regulates cell proliferation and differentiation [70]. The secreted Wnt family of proteins directs cell fate determination at various stages of development [71,72]. The human Wnt gene family consists of 19 highly conserved cysteine-rich glycoproteins [73]. The physiological response elicited by Wnt proteins when they bind to the transmembrane receptor Frizzled (Fzd) is used to classify them. Wnt proteins that induce a β-catenin-dependent transcriptional response are referred to as “canonical”, whereas those that induce alternative responses are referred to as “non-canonical”. The noncanonical Wnt pathway employs a more diverse set of cytoplasmic signaling molecules than the canonical response, making it less well understood [74]. The Wnt/β-catenin signaling cascade is initiated when certain Wnt isoforms bind to their respective Frizzled receptors along with one of the LDL receptor proteins (LRP5 or 6) [75].

The Wnt-Fzd connection recruits a massive cytoplasmic regulatory complex scaffolded by the tumor suppressor proteins, Axin and APC, to the membrane. In normal cells, the proteosome destruction complex destroys β-catenin following its phosphorylation by tGSK-3b, a serine/threonine kinase. Wnt pathway activation stabilizes β-catenin, allowing it to reach the nucleus and influence gene transcription [76,77].

The Wnt/-catenin pathway has been linked to TNBC carcinogenesis and treatment resistance [44,45]. Wnt signaling can lead to the activation of the expression of genes involved in angiogenesis, metastasis, and increased survival and proliferation (Figure 1). Nearly 30 phytochemicals are known to affect the Wnt signaling pathway. These phytochemicals belong to various classes of compounds, including flavonoids, glycosides, and polyphenols [78]. Most of these phytochemicals act by targeting various individual components of the pathway, including Axin, β-catenin translocation, GSK-3β, AKT, and Wif-1.

### 3.3. Targeting Pathways Involved in Tissue Remodeling, Angiogenesis, and Metastasis in TNBC

Invasion and metastasis are complicated processes that entail the disruption of normal cellular and tissue connections, followed by cancer cell migration and colonization in distant organs. Several signaling pathways have been linked to the control of invasion and metastasis in TNBC. These include the epithelial-to-mesenchymal transition (EMT) pathway [79,80,81], the integrin pathway [82,83], the matrix metalloproteinase (MMP) system [84,85], and the chemokine pathway [86,87,88]. The establishment of new blood vessels via angiogenesis is another important pathway that can be targeted.

Tumor tissue, like normal tissue, is unable to develop or spread locally or systemically in the absence of angiogenic support. Blood vessels deliver the oxygen and nutrients needed for growth. Endothelial cells divide and move in response to environmental cues to form blood vessel walls. The activation of the endothelial cell wall of an existing tiny blood vessel (capillary), the production of metalloproteinase enzymes that destroy the proteinaceous extracellular matrix (surrounding tissue), invasion of the matrix, and cell division are the sequential phases of new blood vessel formation. Endothelial cells generate new blood vessel networks that support the growth of surrounding cancer tissue [89,90]. Endothelial mesenchymal transition improves the migratory, invasive, and proliferative properties of endothelial cells. Activated proteases (such as MMPs) can then degrade the surrounding ECM and basement membrane, allowing for directed migration and proliferation [91]. The major signaling pathways required for the initial morphogenetic events are VEGF and Notch [92]. Cancer growth and spread necessitate angiogenic support. Angiogenin, epidermal growth factor (EGF), oestrogen, angiostatin, endostatin, interferons, interleukins 1 and 12, tissue inhibitors of metalloproteinases, and retinoic acid are examples of natural angiogenesis inhibitors [89,93,94,95].

**Figure 3:**
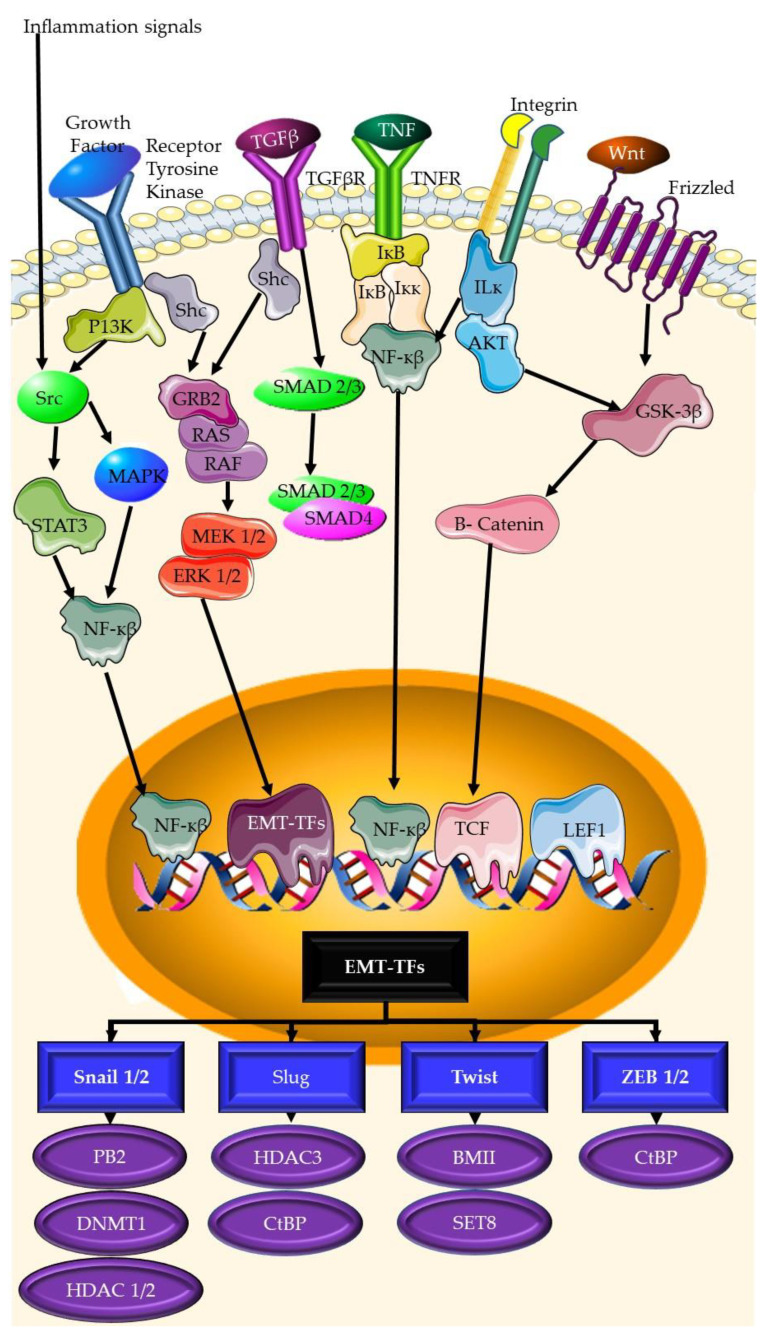
The EMT transition pathway. The initiation of endothelial to mesenchymal transition is required for the spread and invasion of cancer as well as angiogenesis, which is required for the growth of tumors. The EMT pathway can be activated by multiple receptor tyrosine kinases as well as through Wnt, integrin, and inflammation signals. The end result is the activation of what is known as EMT transcription factors (TFs), which lead to the transcription and expression of a multitude of genes that are involved in the EMT process.

The EMT pathway (Figure 3) is the signaling pathway that controls the process in which epithelial cells lose cell-cell connections and adopt a mesenchymal character, resulting in enhanced motility and invasiveness [96]. The EMT pathway has been linked to the development and progression of invasion and metastasis in TNBC, and its activation is linked to a worse prognosis [97,98].

The integrin pathway is involved in the regulation of cell adhesion, migration, and invasion [99,100]. In TNBC, alterations in integrin expression and function have been reported, and they have been implicated in the promotion of invasion and metastasis [101,102].

The MMP pathway participates in the destruction of the extracellular matrix (ECM), which is required for tumor invasion and metastasis [103,104]. MMPs are overexpressed in TNBC and linked to a poor prognosis [105,106]. The chemokine pathway regulates cell motility and is essential for the recruitment of immune cells to the location of a malignancy [107,108,109]. Chemokine expression and function are altered in TNBC, and they have been linked to the stimulation of invasion and metastasis [110,111].

Finally, TNBC is distinguished by its aggressive nature and proclivity for invasion and metastasis. The EMT route, the integrin pathway, the MMP pathway, and the chemokine pathway are all involved in the control of invasion and metastasis in TNBC. A greater knowledge of these pathways may lead to the identification of new targets for therapeutic treatments to prevent or cure TNBC invasion and metastasis.

### 3.4. Phytochemicals Serving as Antioxidants: Cancer Prevention

Oxidative stress can contribute to the development of cancer through the redox states of many regulatory molecules, signal transduction, regulation of enzyme activity, the control of the cell cycle and proliferation, as well as DNA damage [112,113]. The activity of many key transcription factors involved in cell cycle regulation can be modulated by ROS. This mainly occurs through oxidative modifications of specific amino acid residues in the DNA-binding motif of the protein or redox-induced changes in phosphorylation status. Depending on the transcription factor in question, redox modifications can serve to either increase or decrease transcriptional activity [114,115]. Certain oncogenic signals may increase the generation of reactive oxygen species (ROS) due to metabolic activity associated with uncontrolled cell growth and proliferation. The genetic instability associated with ROS-mediated DNA damage may provide a mechanism to render them more vulnerable to further oxidative insults by exogenous ROS-generating agents, thereby forming the basis for therapeutics [116]. Some phytochemicals function as natural antioxidants, neutralizing free radical damage and decreasing the risk of cancer formation in humans [117]. Plant antioxidants include phenols, flavonoids, and polyphenols; tocopherols; and terpenoids [118]. A variety of plant phytochemicals, including terpenoids, phenolic acids, and alkaloids, have been shown to have anticancer properties through fine-tuning reactive oxygen species’ (ROS) signaling pathways [119]. This is consistent with the antioxidative and ROS-scavenging capabilities of several phytochemicals, which have been examined and found to trigger apoptosis via the production of ROS. Xanthohumol, for example, is a widely utilized phytochemical. It is a prenylated flavonoid that was originally identified in hops, *Humulus lupulus* L. [120]. It also acts to inhibit DNA synthesis, induce apoptosis, and prevent cancer cell invasion [120].

### 3.5. Induction of Apoptosis

One of the most common modes of action for phytochemicals in the treatment of cancer is through the induction or modulation of autophagy and apoptosis. Bcl-2 expression and Bax expression have been shown to be critical in promoting the death of breast cancer cells through the release of cytochrome c and inducing the caspase cascade by the phytochemical Pyranocycloartobiloxanthone A [121]. Capsaicin, a significant phytochemical, has been shown to trigger apoptosis in a variety of cancer cells [122]. A number of other phytochemicals have been reported to be useful in cancer treatment by reducing NF-kB activity and stimulating cancer cell death [6]. In cancer cells, NF-kB expression is always high because of its involvement in regulating anti-apoptotic and apoptotic genes [123]. By targeting the NF-kB signaling pathway, flavonoids have been found to enhance cancer cell death. Examples of this are the prevalent phytochemicals xanthohumol [124], magnolol [125], morusin [126], urosolic acid [127], and corilagin [128]. Flavonoids have been shown to induce apoptosis in cancer cells by selectively targeting the NF-kB signaling pathway.

## 4. Current Treatments for Triple Negative Breast Cancer: An Overview

TNBC is frequently treated surgically, with options including lumpectomy or mastectomy [129]. Radiation treatment, in combination with surgery, may be utilized to lower the chance of recurrence in surrounding tissue [130]. Chemotherapy is a popular adjuvant treatment that includes taxanes (paclitaxel and docetaxel) [131], platinum compound (cisplatin) [132], and anthracycline (doxorubicin) [133]. These drugs work by targeting and killing rapidly dividing cells, such as cancer cells, but they can also harm healthy cells. These disrupt the cell cycle, causing DNA damage and ultimately cell death, causing nausea, hair loss, and fatigue [134].

In recent years, immunotherapy has emerged as a promising therapeutic option for TNBC [135]. Immunotherapy, particularly checkpoint inhibitors, work by enhancing the immune system’s capacity to recognize and remove cancer cells. Anti-PD-1 and anti-CTLA-4 antibodies, for example, prevent negative regulatory molecules from acting on the surface of immune cells, allowing the immune system to detect and destroy cancer cells [136]. These medications boost the body’s immune system, allowing it to target and eliminate cancer cells [136,137]. However, the response rate to immunotherapy remains low [138] and further studies are needed to establish the best course of action.

Overall, present TNBC treatments have limits and there is a need for the development of new, more effective medicines. The combination of chemotherapy and immunotherapy shows potential for improving TNBC patient outcomes. It is vital to remember that each TNBC patient’s treatment strategy will differ depending on their particular situation and stage of the disease.

## 5. Phytochemicals: An Emerging Therapeutic Option for TNBC

**Figure 4:**
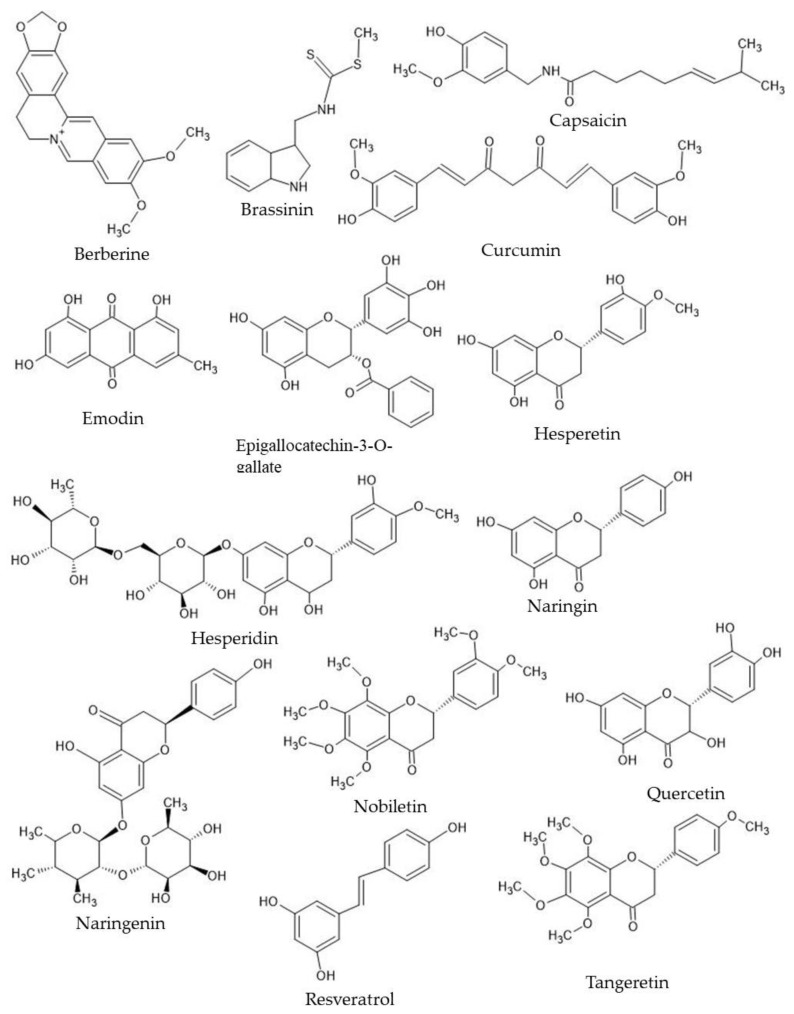
Structure of phytochemicals discussed in this review.

Phytochemicals have recently surfaced as a viable therapy option for TNBC [139]. Phytochemicals are plant-derived natural substances that have been demonstrated to have anticancer activities [140]. They have gained popularity as potential cancer therapies because of their ability to target many signaling pathways and biological processes that contribute to tumor formation and progression [141]. Phytochemicals can fight cancer in a variety of ways, including the induction of apoptosis, the inhibition of angiogenesis, immune system regulation, and cell growth suppression, among others [142]. The phytochemicals discussed in this review are summarized in Table 1 and the structures of these compounds are shown in Figure 4.

Curcumin, a phytochemical isolated from *Curcuma longa*, or turmeric, has already been shown to be effective in the treatment of various cancers by inhibiting cell proliferation and inducing apoptosis [143]. In the treatment of osteosarcoma, curcumin has been shown to target MAPK/ERK, PI3k/AKT, Wnt/β-catenin, Notch, and microRNA [143]. The targeting of the Wnt signaling pathway by curcumin not only inhibits the signaling pathway, but also modifies downstream mediators of the Wnt pathway. These include c-Myc and cyclin D1 [144]. Curcumin also has antioxidant, anti-inflammatory, cardio-protective, hepato-protective, and anti-diabetic activities [144]. Curcumin interrupts cell proliferation, survival, angiogenesis, and metastasis in TNBC. It is able to act on multiple pathways. These include apoptotic and cell cycle pathways, such as the PI3K/Akt/mTOR pathway, the JAK/STAT pathway, the MAPK pathway, the p53 pathway, and the Wnt/β-catenin pathway [145]. In TNBC cells, curcumin has been demonstrated to reduce cell growth, cause apoptosis, and prevent angiogenesis [146,147]. The ability of curcumin to treat TNBC was demonstrated using the hormone-resistant breast cancer MCF-7Ra cell line. This cell line produces aromatase and oestrogen receptor alpha (Erα) while overexpressing the ABCB1 multidrug transporter. Curcumin was able to effectively induce cell death in these cells. Curcumin was able to reduce the expression of genes that drive breast cancer [145,148].

The anticancer phytochemical brassinin is derived from cruciferous vegetables and is active against many different types of cancer. When tested against TNBC, this compound reduces the viability of cells and kills endothelial cells before other tumor cells in vitro. Brassinin has negative effects on angiogenesis in TNBC cells, inhibiting proliferation, migration, tube formation, and spheroid sprouting. When it was regularly administered to a dorsal skinfold chamber model of TNBC, it reduced tumor size, microvessel density, and the perfusion of tumor microvessels. The molecular basis of the activities of brassinin include promoting the degradation of Tie2 and fibroblast growth factor receptor 1 [149].

Resveratrol, found in red grapes and red wine, is one of the most well-known anticancer compounds [150,151]. Resveratrol has been found to cause apoptosis, decrease angiogenesis in TNBC cells, and modify the immune system’s response to TNBC tissue and cells [152,153].

Berberine, is an isoquinoline plant-derived alkaloid, was reported to have anticancer activities, resulting in the creation of synthetic 13-arylalkyl derivatives. Berberine targets the Wnt/β-catenin signaling pathway, inhibiting β-catenin transcriptional activity, and increasing the expression of E-cadherin. Berberine has very low cytotoxicity against normal cells (up to 20 µM). Some of the synthetic compounds based on berberine have higher activity than the natural compound [154]. Another phytochemical that targets Wnt signaling is emodin (1,3,8-trihydroxy-6-methylanthraquinone). This compound is present in the roots and bark of various plants that have a reputation for their medicinal value. These include rhubarb, buckthorn, and Japanese knotweed. It is an anthraquinone and is able to down-regulate Wnt signaling in SW480 and SW620 colorectal cancer cells [155]. The compound inhibits the transcriptional activity of TCF/LEF and down-regulates β-catenin levels. It also affects the expression and activity of various downstream targets of Wnt signaling, such as cyclin D1, c-Myc, snail, vimentin, MMP-2, and MMP-9. Emodin has been observed to stimulate mesenchymal to epithelial transition, increase E-cadherin expression, inhibit migration, and induce growth arrest [155].

Cancer risk can be reduced by increasing intake of fruits, vegetables, and whole grains. The phytochemicals present in these fruits and vegetables can be referred to as “dietary available phytochemicals”. These phytochemicals are able to play anticancer roles by targeting a large variety of cell signaling pathways [156]. Phytochemicals obtained from citrus fruits include naringin, naringenin, tangeretin, nobiletin, hesperidin, and hesperetin, which have been shown to have the ability to inhibit metastasis. These effects have been observed in various cancer cell lines as well as in xenografted mouse models of cancer. These compounds target Wnt, TGF/SMAD, and NOTCH signaling [157].

Other phytochemicals with promising anticancer effects on TNBC cells, in addition to resveratrol and curcumin, are epigallocatechin-3-*O*-gallate (EGCG) found in green tea [158,159,160,161], capsaicin found in chili peppers [162,163,164], and quercetin found in onions [165,166]. In TNBC cells, these phytochemicals have been found to promote apoptosis, block angiogenesis, and affect the immune system.

Despite encouraging preclinical results in TNBC cells, phytochemicals’ promise as a therapeutic option for TNBC has yet to be demonstrated in clinical studies. Only a few clinical studies have been undertaken so far, with inconsistent outcomes. These experiments were hampered by small sample sizes, a lack of standardization, and low phytochemical bioavailability [167,168].

Finally, the molecular mechanisms by which phytochemicals exercise their anticancer effects in TNBC cells are complicated, including various signaling pathways and cellular activities. Phytochemicals have the potential to be a beneficial therapeutic option for TNBC, but further study is needed to confirm their safety and efficacy in clinical studies. The discovery of a safe and effective phytochemical-based therapy for TNBC may give patients a unique and safe choice, enhancing their outcomes.

**Table 1:** Characteristics of phytochemicals discussed in this review.

Compound	Origin	Formula	Ref
Catechin			
Epigallocatechin-3-*O*-gallate	Green Tea	C_22_H_18_O_11_	[158]
Capsaicinoid			
Capsaicin	Chili Peppers	C_18_H_27_NO_3_	[162,163,164]
Dithiocarbamic Ester and an Indole Phytoalexin			
Brassinin	Vegetables	C_11_H_12_N_2_S_2_	[149]
Flavanoid			
Hesperidin	Citrus Fruit	C_28_H_34_O_15_	[156]
Hesperetin		C_16_H_14_O_6_	
Naringenin		C_15_H_12_O_5_	
Naringin		C_27_H_32_O_14_	
Nobiletin		C_21_H_22_O_8_	
Quercetin	Onion	C_15_H_10_O_7_	[165,166]
Tangeretin	Citrus Fruit	C_20_H_20_O_7_	[156]
Isoquinoline Aalkaloid)			
Berberine	Berries	C_20_H_18_NO_4_	[154]
emodin (1,3,8-trihydroxy-6-methylanthraquinone).	Rhubarb, Buckthorn, Japanese Knotweed	C_15_H_10_O_5_	[155]
Phenols			
Curcumin	*Curcuma Longa*	C_21_H_20_O_6_	[143]
Stillbenoid			
Resveratrol	Red Grapes	C_14_H_12_O_3_	[152,153]

## 6. Phytochemicals for TNBC: Limitations and Future Directions

Despite phytochemicals’ promising anticancer properties, such as decreasing cellular proliferation, inducing apoptosis, and modifying signal transduction pathways, their use as a TNBC treatment has a number of problems.

The heterogeneity of treatment effectiveness is one of the most significant constraints. The impact of phytochemicals varies substantially depending on the kind of phytochemical and the individual TNBC subtype, making determining the ideal dose and delivery protocol problematic [169]. While resveratrol has been proven in vitro to suppress TNBC cell growth, its impact in animal models and human studies has been variable [170].

Another drawback is a lack of understanding of phytochemical pharmacokinetics and pharmacodynamics. This lack of understanding makes predicting their efficacy and safety in the clinic difficult, as well as causing difficulties in determining potential interactions with other medications that might influence the overall result of the therapy [171,172,173].

Furthermore, phytochemical sources frequently vary greatly in terms of content and quality. This diversity can make standardizing phytochemical-based therapies and ensuring their consistent therapeutic effectiveness difficult. The composition of plant extracts, for example, might change based on factors such as growing conditions, season, and harvesting method, which can impact the concentration and stability of phytochemicals [174].

To summarize, while phytochemicals show tremendous potential for targeting TNBC pathways, there are numerous limitations that must be addressed before they can be extensively used as a therapy option. More research is needed to learn more about the pharmacokinetics and pharmacodynamics of phytochemicals and to find ways to make their production and use more uniform.

## 7. Conclusions

To summarize, triple negative breast cancer (TNBC) is a very aggressive type of breast cancer with few therapeutic choices. Chemotherapy and radiation therapy, which are currently used to treat TNBC, can have serious adverse effects and are not always successful. Plant-derived phytochemicals, which are physiologically active substances, have shown potential as an alternate therapy for TNBC. TNBC signaling pathways are complicated, involving growth factor receptors, signal transduction pathways, and cell cycle regulators.

**Figure 5:**
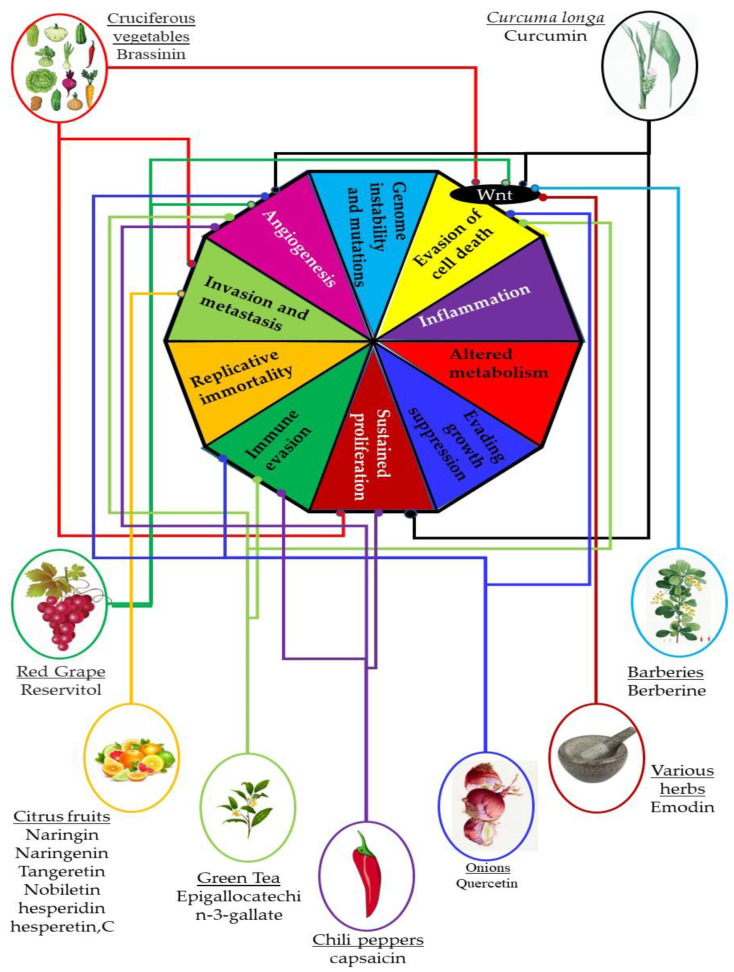
A summary of the targeting of cancer pathways by phytochemicals. Multiple phytochemicals isolated from multiple different species and families of plants act on what are termed the hallmarks of cancer. These are the processes that define the transition from normal cells to cancer cells. Despite these compounds being derived from a wide range of plants, they target very common pathways. These include immune evasion, angiogenesis, metastasis, proliferation, and the inhibition of cell death.

Phytochemicals have been used for cancer treatment since ancient times, with several traditional medical systems employing plants and their extracts to cure cancer. Numerous studies have recently demonstrated the usefulness of phytochemicals in the treatment of TNBC, including curcumin, resveratrol, and green tea polyphenols (Figure 5). These phytochemicals have been shown to target particular signaling pathways in TNBC and suppress cancer cell proliferation. In numerous cases, a variety of phytochemicals exhibit the ability to target several common hallmarks of cancer, with many of them utilizing the same biological pathways for their anticancer effects (Figure 5).

The various plant-derived phytochemicals mentioned in this paper target the different pathways involved in the hallmarks of cancer. These chemicals clearly target very similar pathways. However, the use of phytochemicals as a therapy for TNBC is still in its early phases, and there are several restrictions to be aware of. The scarcity of standardized and high-quality phytochemical extracts, as well as the need for further study to discover appropriate doses and the most effective phytochemical combinations, are key challenges. Even with these problems, the possible benefits of phytochemicals in treating TNBC are important enough to warrant more research and clinical studies.

## Data Availability

Not applicable.
